# Type I interferon regulates cytokine‐delayed neutrophil apoptosis, reactive oxygen species production and chemokine expression

**DOI:** 10.1111/cei.13525

**Published:** 2020-10-15

**Authors:** L. Glennon‐Alty, R. J. Moots, S. W. Edwards, H. L. Wright

**Affiliations:** ^1^ Institute of Life Course and Medical Sciences University of Liverpool Liverpool, Merseyside UK; ^2^ Liverpool Health Partners University of Liverpool Liverpool, Merseyside UK; ^3^ Department of Rheumatology Aintree University Hospital Liverpool UK; ^4^ Institute of Infection, Veterinary and Ecological Science University of Liverpool Liverpool, Merseyside UK

**Keywords:** apoptosis, interferon alpha, neutrophil, p38 MAPK, ROS

## Abstract

Interferons (IFNs) are key regulators of a number of inflammatory conditions in which neutrophils play an important role in pathology, such as rheumatoid arthritis (RA) and systemic lupus erythematosus (SLE), where type I IFNs are implicated in disease pathology. However, IFNs are usually generated *in vivo* together with other cytokines that also have immunoregulatory functions, but such interactions are poorly defined experimentally. We measured the effects of type I (IFN‐α) IFN, elevated in both RA and SLE, on the functions of healthy neutrophils incubated *in vitro* in the absence and presence of proinflammatory cytokines typically elevated in inflammatory diseases [tumour necrosis factor (TNF‐α), granulocyte–macrophage colony‐stimulating factor (GM‐CSF)]. IFN‐α alone had no effect on neutrophil apoptosis; however, it abrogated the anti‐apoptotic effect of GM‐CSF (18 h, *P* < 0·01). The enhanced stability of the anti‐apoptotic protein myeloid cell leukaemia 1 (Mcl‐1) and delayed activation of caspase activation normally regulated by GM‐CSF were blocked by IFN‐α: this effect was mediated, in part, by activation of p38 mitogen‐activated protein kinase (MAPK). IFN‐α alone also primed reactive oxygen species (ROS) production and maintained the transient priming effect of TNF‐α for up to 4 h: it also down‐regulated GM‐CSF‐ and TNF‐α‐activated expression of chemokine (C‐X‐C motif) ligand (CXCL)1, CXCL2, CXCL3, CXCL8, CCL3 and CCL4 but, in contrast, increased the expression of CXCL10. These novel data identify complex regulatory signalling networks in which type I IFNs profoundly alter the response of neutrophils to inflammatory cytokines. This is likely to have important consequences *in vivo* and may explain the complexity and heterogeneity of inflammatory diseases such as RA, in which multiple cytokine cascades have been activated.

## Graphical abstract

Type‐I interferon regulates cytokine‐delayed neutrophil apoptosis, reactive oxygen species production and chemokine expression. This is mediated in part via activation of p38 MAPK.

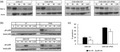

## Introduction

Interferons (IFNs) are a family of cytokine glycoproteins that regulate the immune response to infection by bacteria, viruses and parasites and are broadly grouped into types I, II and III, based on their receptor signalling [1, 2]. Type I IFNs, including interferon (IFN)‐α, IFN‐β, IFN‐ε, IFN‐κ and IFN‐ω, signal through the IFN‐α receptor dimer (IFNAR1 and IFNAR2), whereas type II IFN, IFN‐γ, signals via the IFN‐γ receptor dimer (IFNGR1 and IFNGR2) [1]. Type III IFNs, IFN‐λ1, IFN‐λ2 and IFN‐λ3 [also known as interleukin (IL)‐29, IL‐28α and IL‐28β, respectively] signal via a receptor complex comprising IL‐10R2 and IFN‐LR1 [2]. Interferon receptors signal through the Janus kinase (JAK) family of enzymes [JAK1, JAK2, JAK3, tyrosine kinase 2 (Tyk2)] which, when activated, induce phosphorylation and nuclear translocation of signal transducer and activation of transcription (STAT) proteins [1, 2, 3]. Type I interferons activate heterodimers of JAK1/Tyk2 via activation of the IFN‐α receptor complex leading to activation of STAT‐1/STAT‐2 heterodimers. In some instances, type I IFNs can also activate STAT‐3, STAT‐4 and STAT‐5 [1]. Type II interferons activate heterodimers of JAK1/JAK2 leading to activation of STAT‐1 homodimers.

As well as playing key roles in the host response to pathogen‐mediated infections, IFNs are also implicated in the pathogenesis of a number of inflammatory disorders, including rheumatoid arthritis (RA), systemic lupus erythematosus (SLE), Sjögren’s syndrome and myositis [4, 5, 6]. In SLE, type I interferon signalling correlates with disease severity, and may reflect activation of plasmacytoid dendritic cells (pDCs), which contribute to autoantibody formation in this disease [7, 8]. However, the role of IFNs in RA is less well understood, and tumour necrosis factor (TNF)‐α has generally been considered to be the predominant cytokine that drives disease progression, based on the success of therapeutic TNF‐α blockade [TNF inhibitor (TNF‐i)] [9, 10]. However, there are several reports of an IFN gene expression signature in RA peripheral blood [11, 12, 13, 14], and we recently identified a correlation between a type I ‘IFN‐high’ gene expression signature in peripheral blood neutrophils and good response to TNF‐i in a cohort of RA patients with severe disease activity [4, 15]. As TNF‐α inhibits the activity of pDCs and thereby the production of IFN‐α [16], it appears paradoxical that patients who respond to TNF‐i therapy have high levels of IFN‐regulated gene activity. However, while TNF‐α can often be detected at high levels in RA synovial fluid [17], concentrations of TNF‐α in RA serum are typically low or even undetectable, even in patients who subsequently respond well to TNF‐i therapy [17, 18].

The effect of type II IFN (IFN‐γ) alone on neutrophils has been relatively well defined, and *in vitro* can enhance expression of the high‐affinity cell‐surface molecules FcγRI (CD64) [19] and major histocompatibility complex (MHC)‐II [20], both of which can be detected on the surface of *ex‐vivo* neutrophils isolated from RA synovial fluid [19, 20]. IFN‐γ also delays neutrophil apoptosis and primes the respiratory burst [21, 22]. However, the effect of type I IFNs on neutrophil function is less clear, with studies reporting contradictory effects on both apoptosis and reactive oxygen species (ROS) production via the respiratory burst [21, 23, 24].

The aim of this study was to characterize the functional effects of type I IFN (IFN‐α) on healthy neutrophils in the absence and presence of granulocyte–macrophage colony‐stimulating factor (GM‐CSF) and TNF‐α: cytokines typically elevated in RA. We demonstrate that co‐incubation of healthy neutrophils with IFN‐α profoundly alters their functional responses in ways that have important consequences for understanding neutrophil phenotypes in inflammatory disease, including the blocking of many proinflammatory effects of GM‐CSF. These data reveal potential complex regulatory cytokine‐signalling networks that control immune function in inflammation and inflammatory disease.

## Materials and methods

### Isolation of neutrophils

Participants gave written informed consent according to the Declaration of Helsinki and ethics approval for this study was obtained from the University of Liverpool Committee on Research Ethics. Neutrophils (purity > 97%, viability > 98%) were isolated from healthy heparinized peripheral blood using HetaSep (StemCell Technologies, Cambridge, UK) and Ficoll‐Paque (GE Healthcare, Chicago, IL, USA), and contaminating erythrocytes were removed by hypotonic lysis as described previously [25]. Freshly isolated neutrophils were suspended at 10^6^ or 5 × 10^6^ cells/ml in RPMI media (containing 10% human AB serum; Sigma, St Louis, MO, USA) and incubated at 37°C and 5% CO_2_ for up to 18 h, as indicated in the text. Cytokines were added at the following concentrations: GM‐CSF (5 ng/ml; Roche, Welwyn Garden City, UK), TNF‐α (10 ng/ml; Calbiochem/Fisher Scientific UK Ltd, Loughborough, Gillingham, UK), IFN‐αA2 (0·1–20 ng/ml; Sigma), IFN‐β1a (0·1–20 ng/ml; ImmunoTools, Friesoythe, Germany). Preliminary experiments showed similar results using both type I IFNs (IFN‐αA2 and IFN‐β1a); therefore, IFN‐αA2 was used throughout and is referred to in the text as IFN‐α. Inhibitors of cell signalling were added 30 min prior to cytokine stimulation: BIRB796 [10 μM, p38 mitogen‐activated protein kinase (MAPK) inhibitor; Tocris, Bristol, UK], PD98059 [50 μM, extracellular signal regulated kinase (ERK1/2 inhibitor); Merck, Feltham, ]Gillingham, UK and JNK‐IN‐8 (1 μM, JNK inhibitor; Selleck Chemicals, Cambridge, UK). Cycloheximide (inhibitor of protein synthesis) was used at 10 μg/ml (Sigma) and added 30 min prior to cytokine treatment of neutrophils. Vehicle control (VC) was dimethylsulphoxide (DMSO, Sigma) and was used at the same volume as each stimulant, as indicated in the Figure legends.

### Measurement of apoptosis

Neutrophils (2·5 × 10^4^) were diluted in 50 μl of Hanks’s balanced salt solution (HBSS) (gibco/Thermo Fisher, Waltham, MA) containing 0·5 μl annexin V‐fluorescein isothiocyanate (FITC; (Invitrogen, Inchinnan, Thermo Fisher), and incubated in the dark at room temperature for 15 min. The total volume was then made up to 250 μl with HBSS containing propidium iodide (PI, 1 μg/ml, Sigma) before analysis by flow cytometry (> 5000 events analysed) using a Guava EasyCyte instrument. Apoptosis is reported in the Results section as percentage of annexin V/PI‐positive cells.

### Western blotting

Neutrophils were rapidly lysed in boiling Laemmli buffer containing phosphatase inhibitors (Calbiochem, Merck). Proteins were separated by sodium dodecyl sulphate–polyacrylamide gel electrophoresis (SDS‐PAGE) on a 12% gel and transferred onto polyvinylidene difluoride (PVDF) membrane (Millipore, Watford, Merck). Primary antibodies were: Mcl‐1 (1 : 1000; Cell Signaling, London, UK), caspase‐8 (1 : 1000; Cell Signaling) caspase‐9 (1 : 1000, Cell Signaling), phosphorylated p38 MAPK (1 : 1000; Cell Signaling), total p38 MAPK (1 : 1000; Cell Signaling) and actin (1 : 10 000; Abcam, Cambridge, UK). Secondary antibodies were anti‐rabbit immunoglobulin (Ig)G (GE Healthcare) and anti‐mouse IgG (Sigma) horseradish peroxidase (HRP)‐linked antibodies (1 : 10 000). Bound antibodies were detected using the ECL system (Millipore) on carefully exposed film to avoid saturation (Amersham, Little Chalfont, UK).

### Quantitative polymerase chain reaction (qPCR)

RNA was isolated using Trizol chloroform (Invitrogen, Thermo Fisher), precipitated in isopropanol and cleaned using the RNeasy kit (Qiagen, Manchester, UK), including a DNase digestion step. cDNA was synthesized using the Superscript III first‐strand cDNA synthesis kit (Invitrogen, Thermo Fisher) using equal concentrations of RNA throughout samples, as per the manufacturer’s instructions. Real‐time PCR analysis was carried out using the QuantiTect SYBR Green PCR kit (Qiagen), as per the manufacturer’s instructions. Analysis was carried out on a Roche 480 LightCycler in a 96‐well plate using a 20 μl reaction volume. Primers were as follows: chemokine (C‐X‐C motif) ligand (CXCL)1 forward: TGGCTTAGA ACAAAGGGGCTTA, CXCL1 reverse: AAAGGTAGCCCTTGTTT CCC; CXCL2 forward: TTTCACAGTGTGTGGTCAACAT, CXCL2 reverse: ACACAGAGGGAAACAC TGCATA; CXCL3 forward: ACCGAAG TCATAGCCACACTC, CXCL3 reverse: ACGCTGATAAGCTTCTTACTTCT; CCL3 forward: GCTCTCTGCAACCAGTTCTCT, CCL3 reverse: TGGCTGCTCGTCTCAAAGTAG; CCL4 forward: GCTGTGGTATTCCAAACCAAAA GAA, CCL4 reverse: AGGTGAC CTTCCCTGAAGACT; CXCL8 forward: AAAAGCCACC GGAGCACTCCAT, CXCL8 reverse: AGAGCCACGGCCAGCTTGGA; CXCL10 forward: CCACGTGTTGAGATCATTGCT, CXCL10 reverse: TGCATCGATTTTGCTCCCCT; B2M forward: ACTGAATTCACCCC CACTGA, B2M reverse: CCTCCATGATGCTGCTT ACA. Target gene expression was quantified against B2M as a housekeeping gene using the ∆∆Ct method, normalizing to untreated cells [26, 27]. We also carried out an RNA‐seq experiment (*n* = 1) of neutrophils treated for 1 h with GM‐CSF or TNF ± IFN‐α to identify targets for subsequent validation by qPCR and Western blot. This was carried out using standard RNA‐seq protocols on the Illumina HiSeq 2000 platform, as previously described [15, 25, 28].

### Measurement of the respiratory burst

Neutrophils (5 × 10^6^/ml) were incubated for up to 4 h with IFN‐α (10 ng/ml) in the absence or presence of GM‐CSF (5 ng/ml) or TNF‐α (10 ng/ml). At hourly time‐points, aliquots of cells (2 × 10^5^) were removed from the incubation mixture, diluted in HBSS containing luminol (10 μM, Sigma) and the respiratory burst was stimulated with f‐Met‐Leu‐Phe (fMLP, Sigma) (1 μM). Luminescence was measured continuously, in duplicate, for 15 min in a luminescence plate reader. Total ROS production was measured as area under the curve (AUC), and normalized to no cytokine‐treated cells.

### Statistical analysis

Statistical analysis was carried out using spss version 20, using Student’s *t*‐test unless otherwise stated.

## Results

### IFN‐α abrogates GM‐CSF delayed neutrophil apoptosis via p38 MAPK signalling

**Fig. 1:**
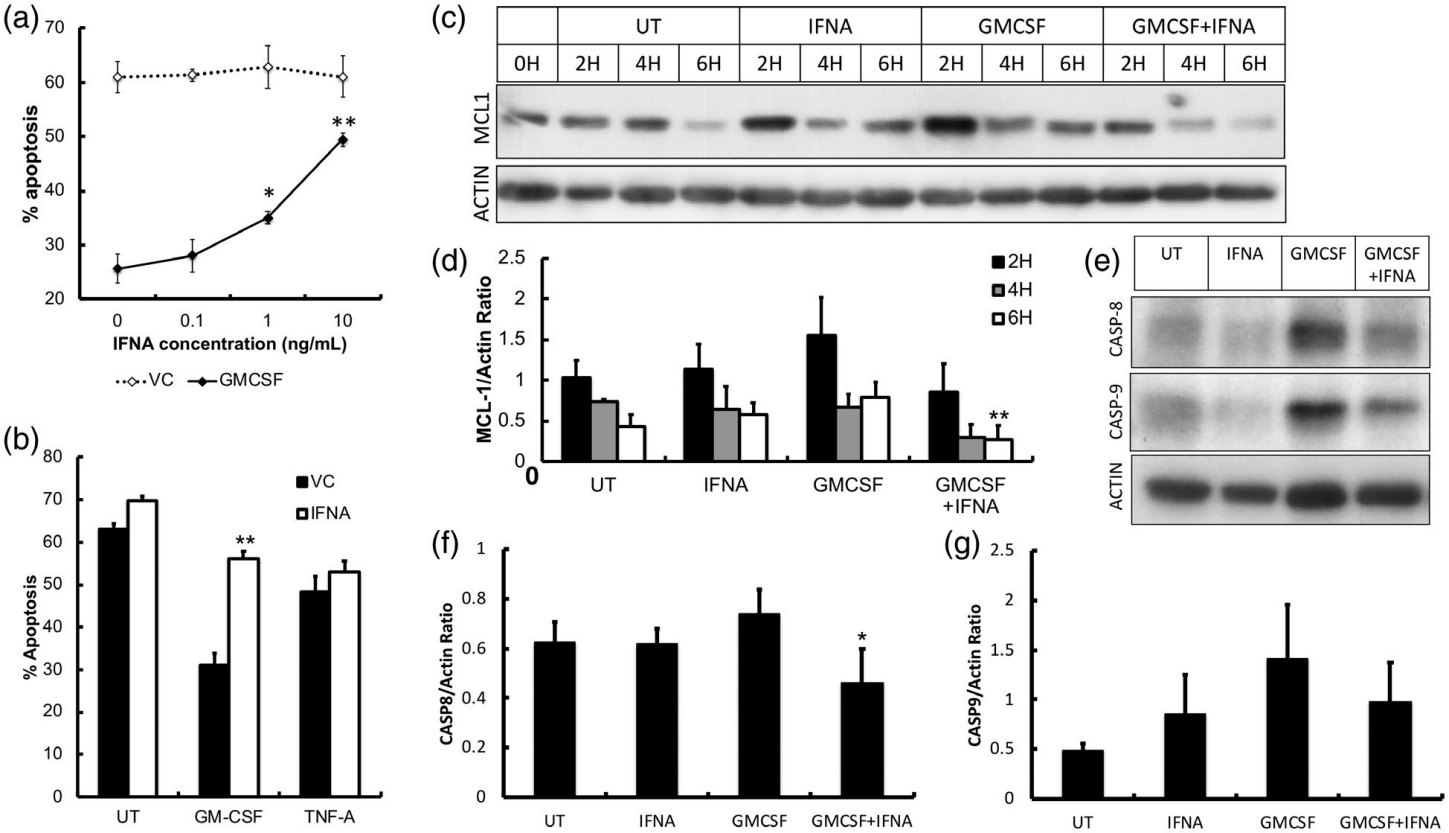
The effect of interferon (IFN)‐α on neutrophil apoptosis. (a) IFN‐α (IFNA) alone had no effect on neutrophil apoptosis over a range of physiologically relevant concentrations (0·1–20 ng/ml) compared to untreated neutrophils (VC = vehicle control) at 18 h. However, IFN‐α abrogated the anti‐apoptotic effect of granulocyte–macrophage colony‐stimulating factor (GM‐CSF) (5 ng/ml). (b) IFN‐α had no effect on the anti‐apoptotic effect of tumour necrosis factor (TNF)‐α at 18 h. (c,d) Expression of myeloid cell leukaemia 1 (Mcl‐1) was lower in neutrophils co‐incubated with GM‐CSF and IFN‐α compared to GM‐CSF alone. (e–g) The levels of pro‐caspase‐8 and ‐9 were lower after 18 h culture in the presence of GM‐CSF and IFN‐α compared to GM‐CSF alone (all experiments *n* = 3, *P < 0·05, **P < 0·01).

Blood neutrophils constitutively undergo apoptosis after approximately 24 h, but during inflammation apoptosis can be delayed by cytokines such as TNF‐α and GM‐CSF [25]. In order to determine the effect of IFN‐α on neutrophil apoptosis, healthy neutrophils were incubated for 18 h in the absence or presence of IFN‐α over a range of concentrations. IFN‐α alone did not significantly alter the rate of constitutive neutrophil apoptosis (Fig. 1a), but it dose‐dependently abrogated GM‐CSF‐delayed apoptosis (Fig. 1a, *P* < 0·05). However, this effect was cytokine‐specific, as it had no significant effect on the rate of apoptosis in neutrophils treated for 18 h with TNF‐α (Fig. 1b). In order to explore the mechanisms responsible for the effect of IFN‐α on GM‐CSF‐delayed apoptosis, neutrophils were incubated for up to 18 h with these cytokines and protein lysates were analysed by Western blot. GM‐CSF has previously been shown to delay neutrophil apoptosis through stabilization of the anti‐apoptotic protein Mcl‐1 [29]. We found that the levels of Mcl‐1 after 6 h incubation were significantly lower in neutrophils treated with GM‐CSF and IFN‐α compared to GM‐CSF alone (Fig. 1c,d, *P* < 0·05). IFN‐α treatment alone had no significant effect on Mcl‐1 levels compared to untreated controls (data not shown). Overnight incubations (18 h) of neutrophils treated with GM‐CSF and IFN‐α had significantly lower levels of pro‐caspase‐8 and pro‐caspase‐9 in the presence of IFN‐α compared to GM‐CSF alone (Fig. 1e–g, *P* < 0·05).

**Fig. 2:**
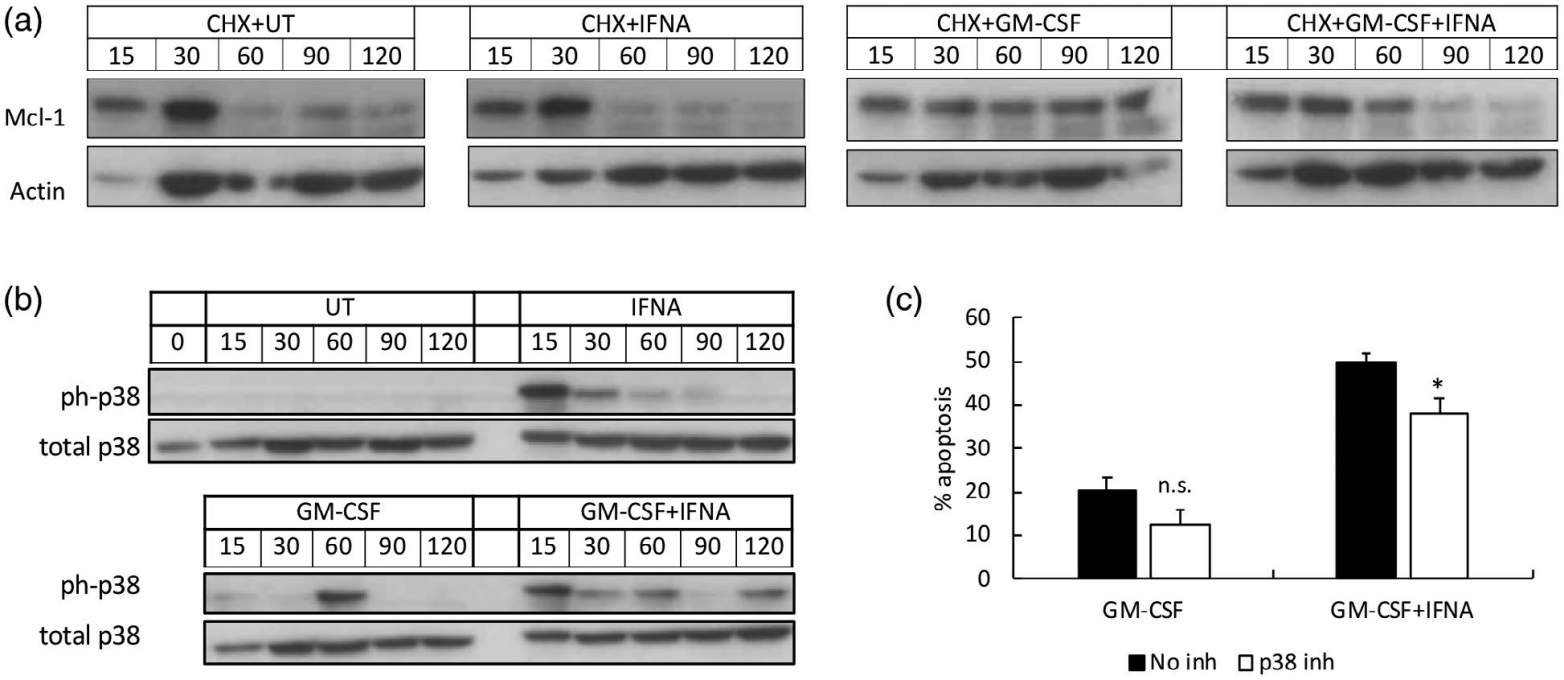
The effect of interferon (IFN‐α) on myeloid cell leukaemia 1 (Mcl‐1) turnover and p38 mitogen‐activated protein kinase (MAPK) activation. (a) Preincubation of neutrophils with cycloheximide prior to granulocyte–macrophage colony‐stimulating factor (GM‐CSF) and IFN‐α showed increased turnover of Mcl‐1 protein levels in the presence of IFN‐α, probably through blockade of Mcl‐1 protein stabilization. (b) p38 MAPK was rapidly phosphorylated by IFN‐α over 120 min in the absence and presence of GM‐CSF. (c) Inhibition of p38 MAPK phosphorylation by BIRB796 significantly inhibited the abrogative effect of IFN‐α on GM‐CSF‐delayed neutrophil apoptosis over 18 h (*n* = 4, *P < 0·05, n.s. = not significant).

Mcl‐1 is an anti‐apoptotic, Bcl‐2 family protein and a key regulator of neutrophil apoptosis. It is rapidly turned over in human neutrophils, and cellular expression of Mcl‐1 correlates with the level of neutrophil apoptosis [30, 31]. However, Mcl‐1 levels can be maintained (and apoptosis delayed) either by phosphorylation and stabilization of existing Mcl‐1 protein or synthesis of new Mcl‐1 protein [29]. Previous work has shown that GM‐CSF stabilizes Mcl‐1 protein, which is largely responsible for its ability to delay neutrophil apoptosis [29]. Preincubation of neutrophils with cycloheximide (CHX), an inhibitor of protein synthesis, prior to the addition of GM‐CSF and IFN‐α, showed that levels of Mcl‐1 protein decreased more rapidly in the presence of GM‐CSF and IFN‐α compared to GM‐CSF alone (Fig. 2a). This suggests that IFN‐α blocks the stabilization of Mcl‐1, probably via interference with post‐translational modification (phosphorylation) of mature Mcl‐1 protein [32]. IFN‐α alone had no effect on Mcl‐1 levels in the presence of CHX (Fig. 2a).

We next investigated the potential mechanism by which IFN‐α abrogates the anti‐apoptotic effect of GM‐CSF using signalling inhibitors of p38 MAPK, JNK and ERK1/2, which are known down‐stream targets of the IFN–AR1/2 complex [1]. While JNK and ERK1/2 were rapidly activated by IFN‐α in the absence and presence of GM‐CSF, inhibition of JNK and ERK1/2 (using JNK‐IN‐8 and PD98059, respectively) had no significant effect on neutrophil apoptosis (data not shown). However, IFN‐α significantly altered the phosphorylation kinetics of p38 MAPK (Fig. 2b), and inhibition of all isoforms of p38 MAPK by BIRB796 significantly decreased the abrogative effect of IFN‐α on GM‐CSF‐delayed apoptosis by approximately 25% (Fig. 2c, *P* < 0·05).

### IFN‐α primes the neutrophil respiratory burst and enhances TNF‐α‐priming

**Fig. 3:**
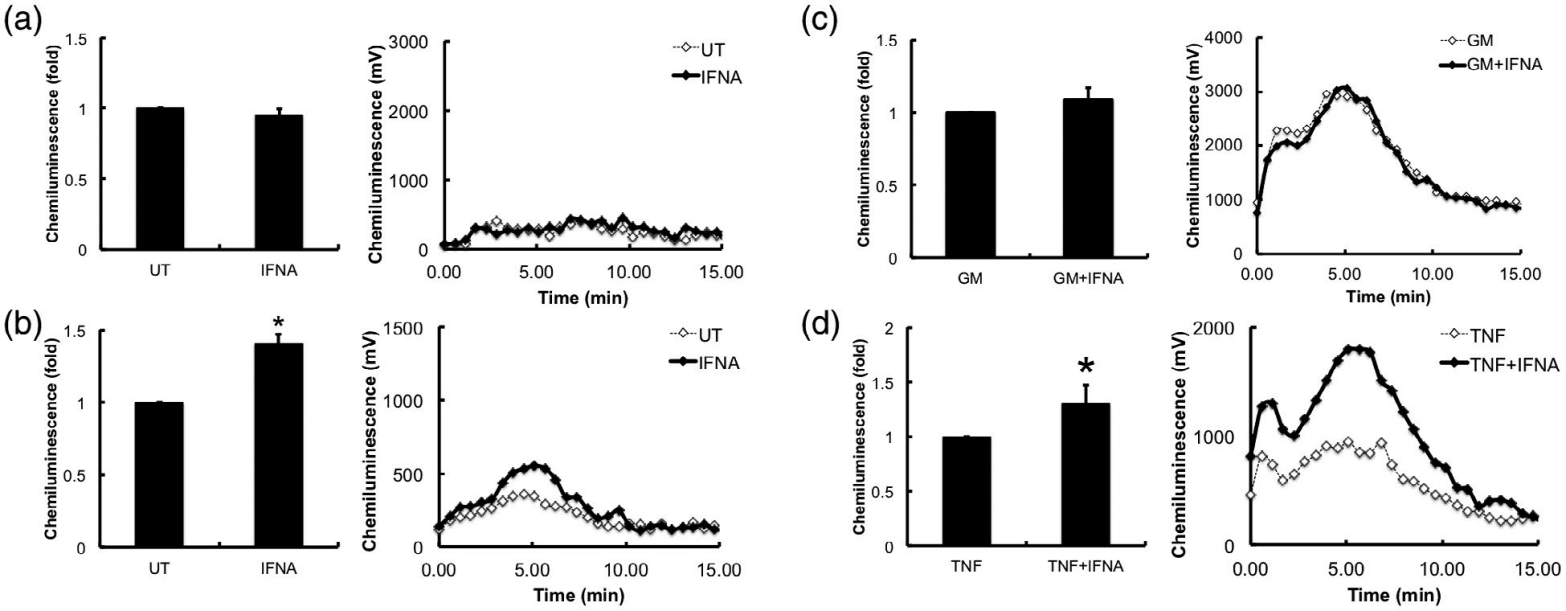
Effect of interferon (IFN‐α) on neutrophil respiratory burst. Neutrophils were incubated with cytokines for 4 h and at hourly time‐points, samples were removed and reactive oxygen species (ROS) production in response to f‐Met‐Leu‐Phe (fMLP) (1 μM) was measured over 15 min. (a) IFN‐α (IFNA) did not prime the respiratory burst in response to fMLP after short incubation times of 1 h, (b) but did significantly prime the respiratory burst after 3 h incubation. (c) Co‐incubation of IFN‐α with granulocyte macrophage–colony‐stimulating factor (GM‐CSF) had no effect on neutrophil priming for up to 4 h. (d) IFN‐α maintained TNF‐induced priming for up to 4 h incubation. Representative traces from four experiments (*P < 0·05, **P < 0·01).

The production of ROS via the respiratory burst is rapidly triggered by the phagocytosis of bacteria [33]. However, in inflammatory diseases such as RA, inappropriate secretion of ROS together with proteolytic enzymes can induce damage to the surface of the joints and degrade cartilage [4, 33]. Using luminol‐enhanced chemiluminescence, we found that neutrophil ROS production in response to fMLP was not primed by 1 h pretreatment with IFN‐α (Fig. 3a). However, ROS production was significantly increased by pretreatment with IFN‐α for 3 h and then stimulated with fMLP (Fig. 3b). Co‐incubation of IFN‐α with GM‐CSF had no significant effect on the GM‐CSF‐primed respiratory burst, which was sustained over a 5‐h incubation period (4‐h time‐point shown in Fig. 3c). TNF‐α rapidly primed the neutrophil respiratory burst over short incubation times of up to 1 h (data not shown), but by 4 h this priming effect was lost. However, co‐incubation with IFN‐α significantly sustained this transient priming effect of TNF‐α over and above the priming effect of IFN‐α alone (Fig. 3d).

### IFN‐α is a key regulator of neutrophil chemokine production

**Fig. 4:**
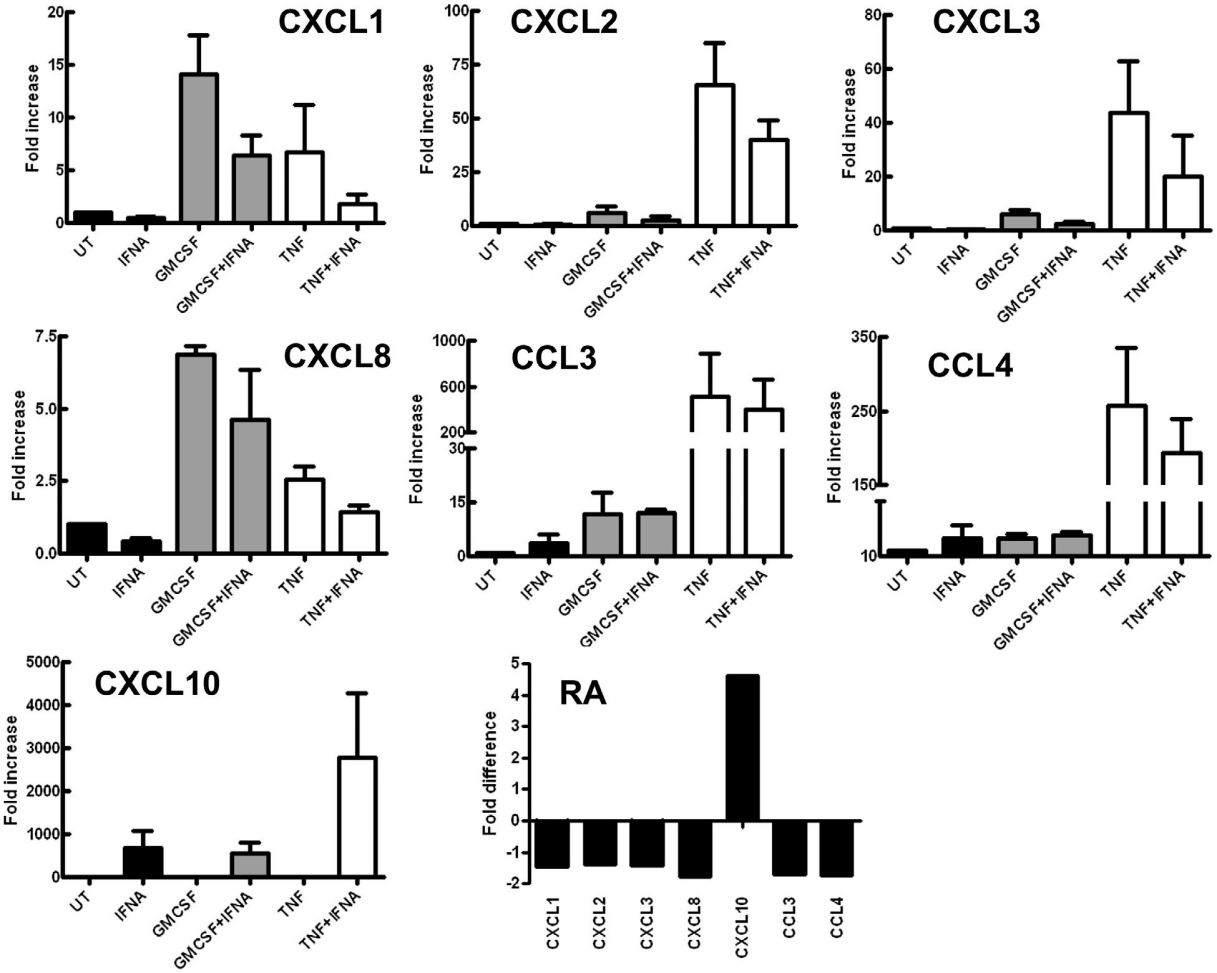
Effect of interferon (IFN‐α) on chemokine expression. Chemokine mRNA expression was measured in neutrophils incubated for 1 h with granulocyte macrophage–colony‐stimulating factor (GM‐CSF) or tumour necrosis factor (TNF)‐α in the absence or presence of IFN‐α (IFNA) (fold change in expression from 0 h shown, three experiments). GM‐CSF and TNF increased expression of chemokine (C‐X‐C motif) ligand (CXCL)1, CXCL2, CXCL3, CXCL8, CCL3 and CCL4. IFN‐α (IFNA) increased expression of CXCL10. Co‐incubation with IFN‐α (IFNA) abrogated the increased expression of chemokines by GM‐CSF and TNF‐α. (e) Rheumatoid arthritis (RA) patients with an IFN‐high gene expression profile expressed chemokines (CXCL1, CXCL2, CXCL3, CXCL8, CCL3, CCL4) at lower levels than IFN‐low RA patients (*n* = 14) levels (fold difference in mean expression level between the two groups of patients shown). The expression level of CXCL10 was higher in IFN‐high RA patients than in IFN‐low patients.

Neutrophils play an important role in the cross‐talk between the innate and adaptive immune system via the secretion of chemokines [34]. We measured the expression of chemokines by healthy neutrophils in response to incubation for 1 h with GM‐CSF and TNF‐α in the absence and presence of IFN‐α using RNA‐Seq (*n* = 1, data not shown), and from these data we selected a panel of genes that were subsequently analysed by qPCR (*n* = 3). GM‐CSF and TNF‐α significantly up‐regulated the expression of CCL3, CCL4, CXCL1, CXCL2, CXCL3 and CXCL8, but not CXCL10 (Fig. 4): GM‐CSF stimulated higher expression of CXCL1 and CXCL8 compared to TNF‐α‐stimulated levels, whereas TNF‐α stimulated higher levels of expression of CXCL2, CXCL3, CCL3 and CCL4. IFN‐α alone significantly up‐regulated the expression of CXCL10 compared to untreated neutrophils (Fig. 4). However, when neutrophils were co‐incubated with IFN‐α and GM‐CSF or TNF‐α, the increase in expression of CCL3, CCL4, CXCL1, CXCL2, CXCL3 and CXCL8 was abrogated (Fig. 4). The expression of CXCL10 by IFN‐α was enhanced in the presence of TNF‐α (Fig. 4).

We next investigated chemokine expression in a group of RA patients who had undergone whole transcriptome sequencing as part of a previous study [4]. In this study, patients clustered into two groups with either ‘IFN‐high’ or ‘IFN‐low’ gene expression profiles. When we analysed gene expression levels for CCL3, CCL4, CXCL1, CXCL2, CXCL3 and CXCL8, we found that these were lower in the ‘IFN‐high’ patients than in the ‘IFN‐low’ patients (Fig. 4e), mirroring the effects we observed with healthy neutrophils treated *in vitro* with IFNs in combination with TNF‐α and GM‐CSF. Interestingly, the expression of CXCL10 was higher in the ‘IFN‐high’ patients than in the ‘IFN‐low’ patients, confirming that the *in‐vivo* phenotype of IFN‐exposed neutrophils correlates with that induced by *in‐vitro* exposure to IFNs. These data imply that the *in‐vitro* regulatory effects of IFNs on neutrophil function that we have observed in this study may also occur *in vivo*.

## Discussion

While the vast majority of *in‐vitro* experiments characterize the effects of single pro‐ or anti‐inflammatory cytokines on immune cells, it is evident that during inflammation *in vivo*, multiple cytokines are involved in the regulation of immune function. In this study, we have investigated the effects of type I interferon (IFN‐α) on neutrophil functions in the absence and presence of two proinflammatory cytokines (GM‐CSF, TNF‐α) that are implicated in the pathogenesis of RA. We have demonstrated that IFN‐α profoundly alters the response of neutrophils to these proinflammatory cytokines in ways that have important implications for understanding neutrophil phenotype in disease.

In our experiments, IFN‐α abrogated the anti‐apoptotic effect of GM‐CSF via blocking the stabilization of Mcl‐1 protein and activation of caspases. We found that IFN‐α altered the phosphorylation kinetics of p38 MAPK, and that blockade of p38 MAPK with a highly selective inhibitor (BIRB796) significantly decreased the effect of IFN‐α on GM‐CSF‐delayed apoptosis. p38 MAPK is a known regulator of Mcl‐1 turnover in neutrophils [29], and inhibition of p38 MAPK using BIRB796 has previously been shown to inhibit Mcl‐1 degradation and induction of apoptosis in myeloid cells induced by purvanalol A, another molecule that can abrogate the anti‐apoptotic effect of GM‐CSF in neutrophils [35]. Sodium salicylate also abrogates GM‐CSF‐delayed neutrophil apoptosis via p38 MAPK signalling and Mcl‐1 turnover mechanisms [29]. Mcl‐1 is an anti‐apoptotic Bcl‐2 family protein that is a key regulator of neutrophil survival. Activators of p38 MAPK signalling, such as vitamin D, have been shown to have a pro‐apoptotic effect on neutrophils via regulation of expression of Bcl‐2 and caspase family member genes [36, 37]. While we saw changes in the levels of Mcl‐1, caspase‐8 and caspase‐9 protein in our experiments, we did not see any change in expression of these genes in our RNA‐seq experiment (data not shown), indicating that the effects of IFN‐α regulating Mcl‐1 dependent apoptosis are probably via post‐translational modifications. However, p38 MAPK has also been shown to be anti‐apoptotic in neutrophils via the regulation of caspase phosphorylation and activation [38, 39], and therefore there remain subtleties in the IFN‐α activation of p38 MAPK that have not been completely addressed in our study. Human neutrophils express two of the four known isoforms of p38 MAPK (p38α, p38δ) [40] and it is likely that these different isoforms exert different regulatory effects on neutrophils. In our study, we used the pan p38 MAPK inhibitor BIRB796 to block activity of all isoforms. While this inhibitor resulted in a statistically significant decrease in the ability of IFN‐α to block GM‐CSF‐induced survival, the effect was only partial, indicating that other signalling pathways activated by IFN‐α can also contribute to these effects. It is interesting to note that IFN‐α did not alter the delay in apoptosis induced by TNF‐α in our study. TNF‐α‐delayed apoptosis in human neutrophils is not dependent upon Mcl‐1 levels or stabilization of Mcl‐1 protein; this is mediated via nuclear factor (NF‐κB) activation and synthesis of the Bcl‐2 family protein A1/Bfl‐1 [41].

IFN‐γ has previously been shown to prime the neutrophil respiratory burst, but the role of IFN‐α in this process is less clear, with conflicting reports in the literature of the effect of IFN‐α on neutrophil apoptosis and ROS production [21, 23, 24]. This may be due to the type of IFN‐α isoform that was used in *in‐vitro* experiments. IFN‐α has 13 different isoforms [42], and the isoform used in our experiments was IFN‐αA2, which is the most abundant isoform in humans. In addition, inconsistencies in the measurement of IFNs as units/ml rather than ng/ml often makes direct comparison of experimental conditions difficult. We comprehensively showed that over incubations of up to 1 h IFN‐α was not able to prime the respiratory burst. However, over longer incubations of > 3 h, IFN‐α primed the respiratory burst, both alone and in combination with TNFα. This may indicate the expression (activated by IFN‐α) of a secondary, autocrine signalling molecule that functions to regulate this late primed response.

Our experiments also identified an important role for IFN‐α in the regulation of chemokine production by neutrophils. We showed that IFN‐α significantly decreased the expression of chemokine genes CXCL1, CXCL2, CXCL3, CCL3 and CCL4 in response to GM‐CSF and TNF‐α, but enhanced the expression of CXCL10. This has important implications for neutrophil‐mediated regulation of the immune response *in vivo*. The chemokines CXCL1, CXCL2 and CXCL3 (GRO‐α, ‐β and ‐γ, respectively) and CCL3 and CCL4 (MIP‐1α and ‐1β, respectively) predominantly function as neutrophil chemoattractants, whereas CXCL10 (IP‐10) is primarily a chemoattractant for T cells, monocytes, NK cells and dendritic cells. Therefore, the cellular signalling events regulated by combinations of inflammatory cytokines (GM‐CSF and/or TNF‐α) and IFNs *in vivo* may act as a switch to direct the immune response away from neutrophil‐mediated inflammation towards lymphocyte‐mediated inflammation, emphasizing a key role of neutrophils in the cross‐talk between the innate and adaptive immune systems [34]. Importantly, we noted that the changes in chemokine expression in the presence of IFN‐α mirrored exactly the different phenotypes seen in RA neutrophils [28], underlying the important role of IFN‐α in the regulation and fine‐tuning of neutrophil function in inflammatory disease.

In summary, our data show that the response of human neutrophils to TNF‐α and GM‐CSF is profoundly altered *in vitro* by IFN‐α, whereby IFN‐α acts as a switch to alter the inflammatory neutrophil phenotype by regulating apoptosis, ROS release and chemokine production. This has important consequences for neutrophil inflammatory responses *in vivo*, particularly in complex and heterogeneous inflammatory diseases such as RA in which combinations of these cytokines exist, and may explain why some patients respond better to certain biological therapies than others. These observations may also explain the great heterogeneity in neutrophil function seen in inflammatory diseases (that are associated with differing amounts and types of cytokines) and the difficulty in correlating particular cell functions with concentrations of a particular activating molecule, such as a cytokine. Modelling these complex interactions is problematic, because such interactions cannot be predicted by measuring the effects of these individual components alone on particular cell functions. Our data reveal a new level of control of inflammation by neutrophils.

## Disclosures

The authors declare no conflicts of interest.

## Author contributions

L. G. A. carried out the experiments and analysed the data. H. L. W. designed the research, carried out the experiments, analysed the data and wrote the manuscript. R. J. M. revised the manuscript. S. W. E. analysed the data and revised the manuscript.

## Data Availability

Not applicable.
